# Integrating nutrition and culinary medicine into preclinical medical training

**DOI:** 10.1186/s12909-024-05795-3

**Published:** 2024-09-03

**Authors:** Emily A. Johnston, Maria Torres, Sara Goldgraben, Christopher M. Burns

**Affiliations:** 1 Department of Medicine, NYU Grossman School of Medicine, New York, NY 10016 USA; 2 California Health Sciences University College of Osteopathic Medicine, Clovis, CA 93611 USA; 3 The University of Arizona College of Medicine – Phoenix, Phoenix, AZ 85004 USA

**Keywords:** Nutrition, Culinary medicine, Team-based learning, Prevention, Undergraduate medical education, Registered dietitian nutritionist

## Abstract

**Background:**

Poor nutrition is a leading cause of preventable death, but is inconsistently taught in medical education and inadequately discussed in medical care. To overcome this problem, we developed a hybrid nutrition team-based learning/culinary medicine approach to integrate practical nutrition knowledge and basic cooking skills into the training of future health professionals.

**Methods:**

Nutrition was integrated into the systems-based courses at a college of osteopathic medicine, complemented by culinary medicine sessions based on the Health meets Food curriculum (HmF; culinarymedicine.org). Students participated in the program for one year and two cohorts of students were included in this analysis. Outcomes were measured via online food frequency questionnaire (FFQ, Vioscreen, Viocare, Inc) and surveys administered via Qualtrics online survey software. Diet quality was measured using the Healthy Eating Index (HEI)-2015. Data were analyzed using SAS 9.4.

**Results:**

One hundred and ninety-five first year students completed a baseline FFQ (97.5% response rate). Mean age of students was 26 years, 47% were female (*n* = 92/195). The average BMI of participants was 24.8 kg/m^2^ (range 17-45.4) and the majority of participants reported being active. Seventy-five students (38%) completed an end of year FFQ. Diet quality was poor among students at baseline (*n* = 195; 67.59 (SD 10.54)) and improved slightly but significantly at the end of year 1 (*n* = 75, 69.63 (SD: 12.42), *p* = 0.04). The survey was administered to the second cohort only; 63 students responded (53% response rate). Talking to patients about nutrition was seen as more relevant to future practice among respondents than talking to patients about safe sex, weight, tobacco, alcohol, other substance abuse and domestic violence.

**Conclusions:**

This study evaluated the nutrition and culinary medicine curriculum at a new college of osteopathic medicine. Students rated the program highly and attendance was excellent, even though not required. Student diet quality did not decline over the first year of medical school. Students rated talking to patients about nutrition as highly relevant, providing encouragement that they will do so in future practice. We believe our work shows that nutrition can be integrated into the training of future physicians and that it may pay dividends, particularly with the increasing awareness of the importance of preventive care.

## Background

Poor nutrition is a leading cause of preventable death globally [1] , but it is taught in less than 30% of US medical schools [2] and discussed in less than 10% of US medical office visits [3]. Doctors of Osteopathic Medicine are trained in holistic care, with a focus on self-regulation, self-healing and health maintenance [4] but few colleges of osteopathic medicine (22/ 26 responding DO schools surveyed) meet minimum hours recommended by the National Academy of Sciences for nutrition education [5] and most provide less than half of the recommended amount [6]. In a survey of 257 preclinical osteopathic medical students, 171 participants (67%) felt that nutrition counseling and meal planning were the responsibility of the physician, but only 30 participants (12%) were aware of the current nutrition guidelines and 130 participants (51%) scored below the school’s passing rate (73%) on a nutrition knowledge quiz [7]. A majority of osteopathic medical students go on to practice in primary care [8] and have the opportunity to treat patients over many years. They are thus well situated to implement preventive strategies with patients, particularly recommending healthy dietary choices.

The American Heart Association [9], the European Society for Parenteral and Enteral Nutrition [10] and others have called for increases in training in nutrition for future physicians. The Association of American Medical Colleges (AAMC) and the American Association of Colleges of Osteopathic Medicine (AACOM) have not released similar statements, although the AAMC did endorse a bill to improve nutrition education in medical schools in 2019 [11]. There are many barriers to increasing rates of nutrition in medical education broadly across the country. Few medical schools have nutrition professionals on the faculty, few curricular standards exist for teaching or assessing nutrition in medical education, there is little open time in the medical curriculum and a lack of questions on board exams. A 2019 Harvard Law Report suggested that the Liaison Committee on Medical Education (LCME) amend their accreditation standards to require nutrition education, which would lead to increased rates of nutrition in medical training [12]. However, there has been no change thus far.

Culinary medicine is an evidence-based educational intervention aimed at helping people to access, prepare and consume nutrient rich meals that taste good, are culturally appropriate and help prevent and manage disease [13]. This provides a strategy for integrating practical nutrition knowledge and basic cooking skills into the training of future health professionals to enable them to better advise their patients on eating well. In the Health meets Food (HmF) culinary medicine curriculum, started at Tulane University [14], students complete pre-work, participate in team-based case study activities, and then prepare healthful meals with their teams. The teams present the meals to the rest of the class and then the entire group eats the meal together and completes an informal debriefing session. The HmF coursework is based on the Mediterranean diet, which has been implicated in reductions in cardiovascular disease risk and other positive health outcomes [15].

While CM sessions are often taught in teams, CM is not technically taught in the team-based learning style. Team-based learning (TBL) is defined by the Team-based Learning Collaborative as “an evidence based collaborative learning teaching strategy designed around units of instruction, known as “modules,” that are taught in a three-step cycle: preparation, in-class readiness assurance testing, and application-focused exercise” [16]. We integrated core concepts of biochemical and physiological aspects of nutrition into the curriculum of an osteopathic medical school via team-based learning modules, self-study activities within the systems-based coursework, and through culinary medicine. Students participated in one culinary medicine workshop (CMW) per systems course (approximately once per month), aligning with the systems-based curriculum of the medical school.

We aimed to integrate nutrition content into the preclinical curriculum with related culinary medicine TBL sessions for medical students in their first year of osteopathic medical training. The purpose of this study was to evaluate perceptions and personal dietary habits of first year osteopathic medical students. We hypothesized that students would be more interested in incorporating nutrition and culinary medicine in their future practice and report improved personal diet quality following these sessions.

## Methods

**Fig. 1:**
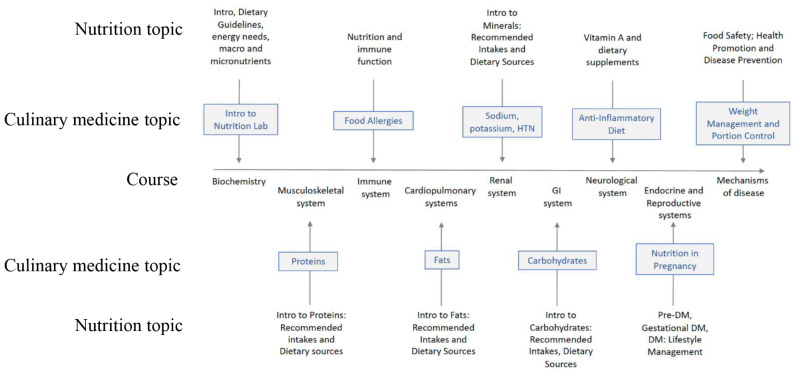
Curriculum outline of nutrition and culinary medicine integration in year 1 of preclinical osteopathic medical training

The nutrition curriculum was created and taught by a nutrition faculty member and Registered Dietitian Nutritionist (RDN). Nutrition was integrated into the systems-based courses as shown in Fig. 1, complemented by culinary medicine sessions on related topics when possible. For example, during the Biochemistry pre-clinical course, the culinary medicine course was Introduction to Nutrition lab, which focuses on safety in the kitchen, basic knife skills, and giving an overview of the program, not on biochemistry. Curricula aligned when possible, such as during the Immune System course when the CM module was Food Allergies. We used the Health meets Food curriculum (HmF; culinarymedicine.org) and selected nine of the available modules to teach the students. Most CMW sessions took place in a teaching kitchen on campus; some sessions were taught virtually due to health and safety requirements resulting from the global pandemic.

Cohort 1 engaged in culinary medicine in the method established by HmF. Cohort 2 engaged in culinary medicine via TBL sessions utilizing the readiness assurance process. Both cohorts were from the same university and were first year medical students. TBL sessions were delivered online via InteDashboard software (CognaLearn Pte. Ltd. Singapore). Two items were dedicated to nutrition coursework on mid-course exams and 2 items were dedicated to culinary medicine on all final exams.

Outcomes were measured via online food frequency questionnaire (FFQ; Vioscreen, Viocare, Inc) and surveys administered via Qualtrics online survey software. Vioscreen provides in-depth reports, including the Healthy Eating Index (HEI-2015) [17], a measure of adherence to the Dietary Guidelines for Americans. Detailed reports from Vioscreen were integrated into future coursework to allow students to apply what they learn in the coursework to their personal diet reports. The Vioscreen FFQ was selected for several reasons, including that it is online and includes pictures to enhance accuracy of portion size estimation. The FFQ includes two questions on multivitamin use: 1. Yes/No; If Yes, how frequently do you take the multivitamin? There is also one question on physical activity where respondents rate their perceived level of activity from sedentary to extremely active. Selected survey questions were adapted from previously published surveys [18].

Data were collected between August 2020 and June 2022. This study was approved by the California Health Sciences University (CHSU) Institutional Review Board. All participants provided written informed consent.

### Data collection and analysis

Students received an email invitation to complete an online FFQ via Vioscreen.com prior to the start of nutrition-related coursework. Dedicated time to complete the questionnaire was allotted as part of the curricular session. Students received several reminders during class sessions to complete the FFQ. Data collection was closed after 1 month. No incentives were offered for participation.

A survey was administered via Qualtrics to Cohort 2 only. The survey asked questions about prior nutrition training, attitudes about relevance of nutrition to medical school coursework, and whether and how medical students access nutrition information.

The survey and FFQ were pilot tested with a group of faculty and staff to identify errors in advance of dissemination to students. No power calculation was performed, all students were invited to complete FFQ’s and surveys. Data were analyzed using SAS 9.4 software. Wilcoxon signed rank test and paired t-tests were used to compare scores from baseline to end of year 1 in those with 2 completed FFQ’s and *p* < 0.05 was used to determine statistical significance.

## Results

One hundred and ninety-five first year medical students completed a baseline food frequency questionnaire (195/200, 98% response rate) and 75 (38%) completed an end of year FFQ. The average age of students was 26 years, 47% of respondents were female (*n* = 92/195). The average BMI of participants was 24.8 kg/m^2^ (range 17-45.4) and the majority of participants reported being active (Table 1).

**Table 1:** Baseline characteristics of study participants (*n* = 195)

Characteristic	
Mean Age (years)	26 (20–42)
Gender (reported)	47% F (92 F, 103 M)
Mean BMI (kg/m^2^)	24.78 (17-45.4)
Multivitamin users*	106 (54%)
Reported Activity Level (n, %)**	
Sedentary	16 (8%)
Low active	72 (37%)
Active	86 (44%)
Very Active	19 (10%)
Extremely Active	2 (1%)

*Respondents who reported they take a multivitamin, regardless of frequency

**Respondents selected their perceived level of physical activity from sedentary-extremely active

### Diet quality

When comparing only participants with 2 complete FFQ’s (*n* = 75) Healthy Eating Index (HEI)-2015 score improved significantly from baseline (2.13, *P* = 0.04). The HEI-2015 Fatty Acid component score was also significantly higher at end of year 1 (Table 2). No other component scores were significantly different between timepoints.

### Nutrient intake

Mean energy intake was 1926 kcals per day (SD: 839 kcals) at baseline and 1512 kcals (SD: 764 kcals) at the end of year 1. Participants reported intake of an average of 23 g/day of fiber per day at baseline and 20 g/day at the end of year 1 (*p* = 0.001). Mean sodium intake was over 3770 mg/day at baseline and 2981 mg/day at the end of year 1 (< 0.001). Mean potassium intake was 2957 mg/day at baseline and 2466 mg/day at the end of year 1 (*p* = 0.0003). Intake in grams and calories of added sugar decreased from baseline to the end of year 1 (both *p* = 0.01). Other individual nutrient scores are listed in Table 3.

**Table 2:** Healthy Eating Index 2015 scores at Baseline vs. End of Year

	Baseline (*n* = 195)		End of Year 1 (*n* = 75)		*P*-value^#^
Variable	Mean	Std Dev	Mean	Std Dev	
HEI-2015 Total Score*	67.59	10.54	69.63	12.42	**0.04**
HEI-2015 Fruit**	3.72	1.51	3.60	1.80	0.98
HEI-2015 Whole Fruit**	4.34	1.28	4.06	1.64	0.34
HEI-2015 Vegetables**	4.15	1.07	4.23	1.11	0.84
HEI-2015 Greens and Beans**	4.09	1.39	4.04	1.57	0.40
HEI-2015 Whole Grains***	4.46	3.17	5.18	3.55	0.14
HEI-2015 Dairy***	6.02	2.64	6.04	2.80	0.80
HEI-2015 Protein Foods**	4.73	0.70	4.73	0.61	0.31
HEI-2015 Seafood and Plant Proteins**	4.40	1.26	4.44	1.24	0.99
HEI-2015 Fatty Acids***	6.37	3.19	6.96	2.96	**0.01**
HEI-2015 Refined Grains***	8.18	2.45	8.47	2.39	0.77
HEI-2015 Sodium***	2.06	2.41	2.31	2.91	0.48
HEI-2015 Saturated Fat***	6.23	3.00	6.83	3.38	0.14
HEI-2015 Added Sugars***	8.84	1.82	8.74	1.94	0.97

*HEI total score out of a maximum of 100; **HEI range: 0–5;***HEI range: 0–10; ^#^Comparisons between 75 students with complete pre and post FFQ data

**Table 3:** Selected nutrient intake at Baseline vs. End of Year

	Baseline (*n* = 196)		End of Year (*n* = 75)^#^		
Variable	Mean	Std Dev	Mean	Std Dev	*P* value
Added Sugars (calories)	42.6	32.9	34.5	37.4	**0.01**
Added Sugars (grams)	9.4	7.2	7.6	7.8	**0.01**
Alcohol (calories)	46.6	137.8	23.7	97.8	**0.03**
Alcohol (servings)	0.4	1.4	0.2	1.0	0.05
Caffeine (mg)	120.7	123.5	99.9	141.3	**0.1**
Fiber (g)	23.4	12.0	20.4	11.5	**0.001**
Potassium (mg)	2957	1338	2466	1289	**0.0003**
Sodium	3770	1862	2981	1826	**< 0.001**

^#^Comparisons between 75 students with complete pre and post FFQ data

### Survey responses

The Qualtrics survey was administered to the second cohort only; 63 students responded (53% response rate; 52% female (*n* = 33)). Respondents reported that they intended to enter primary care fields (19%, *n* = 12), internal medicine (14%, *n* = 9), emergency medicine (13%, *n* = 8), surgery (8%, *n* = 5), psychiatry (6%, *n* = 4), 3% each (*n* = 2 each) selected obstetrics/gynecology and cardiology, 1 student (1.5% each) selected neurology, sports medicine, integrative medicine and pain management; 27% (*n* = 17) were unsure. When asked to rate their current health (*n* = 62 for this item), 15% (*n* = 9) responded that their health is excellent, 40% (*n* = 25) responded very good, 39% (*n* = 24) responded average, and 6% (*n* = 4) responded that their health is poor.

27% of respondents (17/63) reported taking a nutrition class prior to entering medical school and 13% (*n* = 8) reported that they would feel confident assessing a patient’s diet. More than half (*n* = 38) of respondents have done their own reading or research related to nutrition; of those, 58% (*n* = 22) reported getting their information only from diet books or the popular media, 13% (*n* = 5) have read peer-reviewed nutrition journals or nutrition articles geared toward physicians and 26% (*n* = 10) reported getting their nutrition information from both media and peer-reviewed sources (respondents could select more than 1 answer).

60% of respondents (*n* = 38) correctly identified the daily fruit and vegetable recommendations from the Dietary Guidelines for Americans, 51% (*n* = 32) correctly identified the daily fiber intake recommendations, 84% (*n* = 53) correctly identified the added sugar and 43% (*n* = 27) correctly identified the saturated fat recommendations on the knowledge questions based on the Dietary Guidelines for Americans [19].

**Fig. 2:**
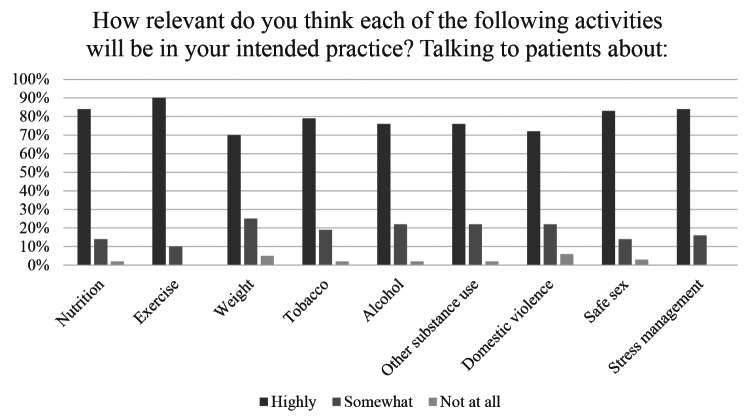
Perceived relevance of prevention topics to intended practice (*n* = 63)

**Fig. 3:**
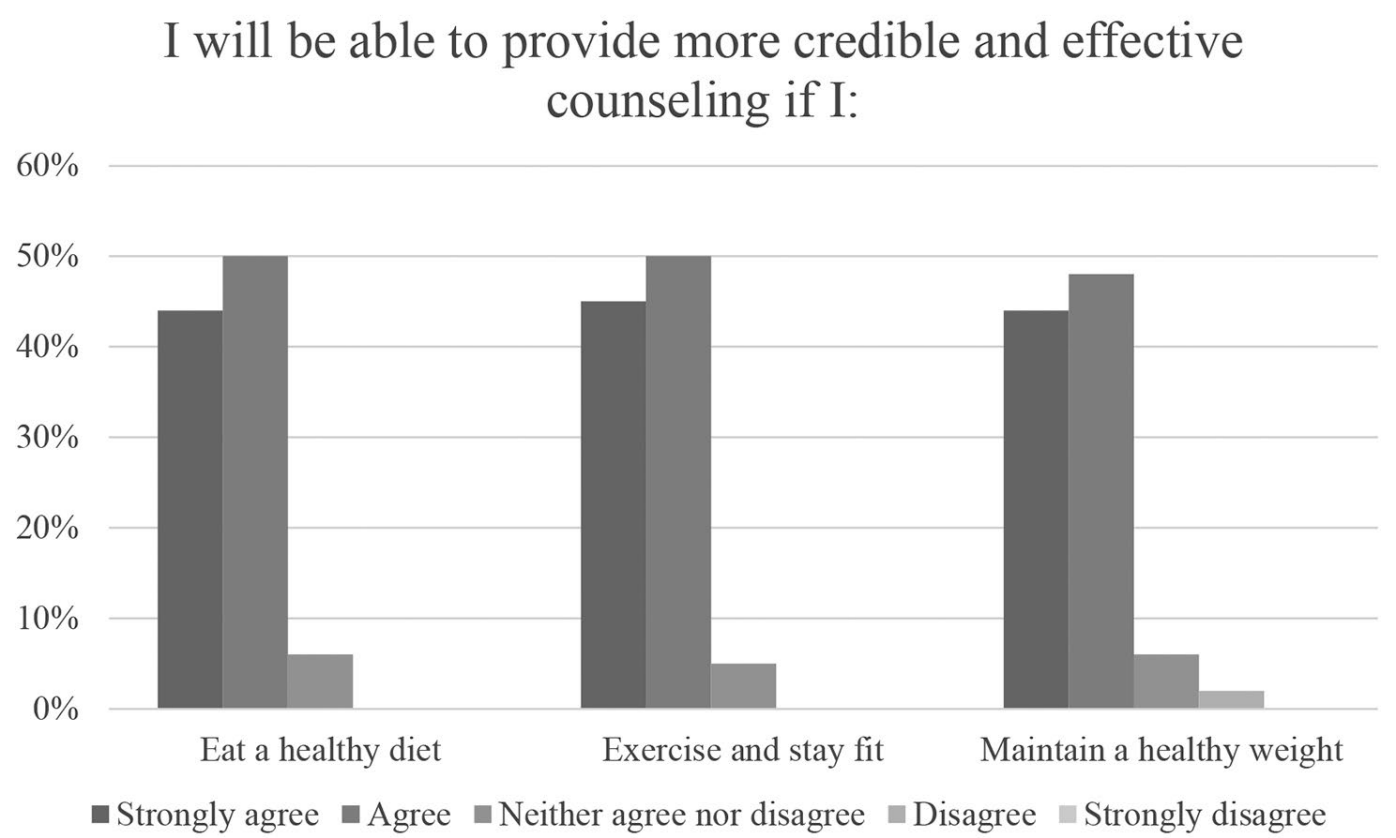
Perceived credibility as a healthcare provider based on personal health habits (*n* = 63)

**Fig. 4:**
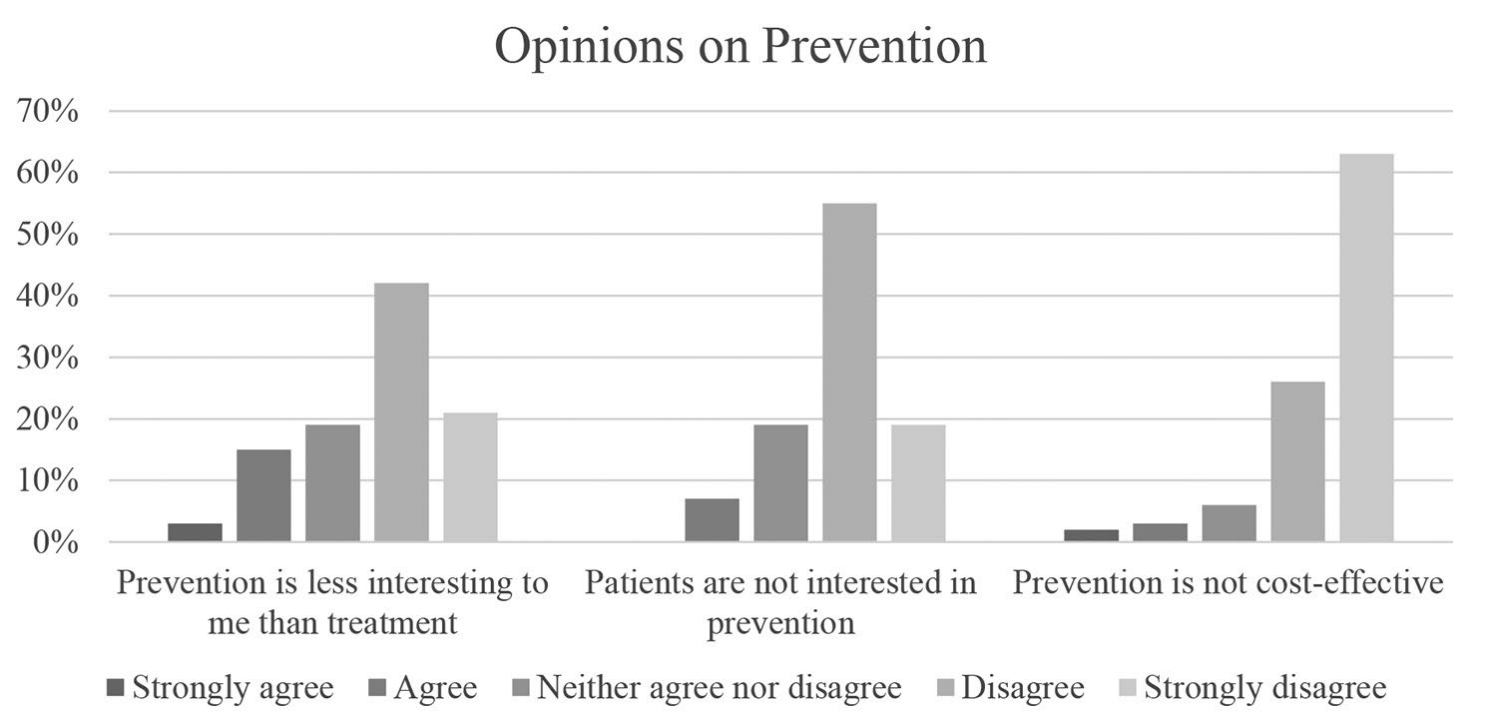
Opinions on prevention topics of medical students (*n* = 63)

84% of respondents (*n* = 53) reported that talking to patients about nutrition is highly relevant to their intended practice, 14% (*n* = 9) rated it somewhat important. Talking to patients about nutrition was more relevant to future practice among respondents than talking to patients about safe sex, weight, tobacco, alcohol, other substance abuse and domestic violence (Fig. 2). Responses to perception questions are displayed in Figs. 2, 3 and 4.

Students favorably reviewed the culinary medicine curriculum, including comments such as: “I love having these classes. It’s such a breather and plus I get to cook!” and

“It [the recipe] was awesome! I will definitely make it again.” Students specifically highlighted eating more legumes and vegetables after participating in culinary medicine (Table 4).

**Table 4:** Student quotes about plant-based meals

“I’ve never been a salad person before this class, now I enjoy eating salads.”
“I add lentils to my spaghetti like we prepared in class, it adds a great flavor.”
“The oven baked veggies were very tasty and a healthier cooking technique that I’d like to incorporate into my life.”
“It [the dish] was good! Never had kidney beans before, and was shocked to see how big they were when I opened the can!”
“I’ve never made this [white bean shakshuka] before. It smells amazing!”

Students also shared stories about how they felt better equipped to include nutrition education in patient care. For instance, after learning about weight management techniques, students shared how they would like to include a discussion of weight management during their future patient interactions.

## Discussion

This study evaluated the nutrition and culinary medicine curriculum at a new college of osteopathic medicine. The culinary medicine curriculum was licensed from an established program (HmF), however the integration with nutrition coursework and the assessment strategies were novel. There was a small but significant change in overall diet quality between baseline and the end of the year among participants who completed food frequency questionnaires at two time points. While students learned about nutrition, they were also adjusting to a new routine, new budget, new environment and new stressors, all of which may have contributed to their dietary choices. Students did not report a decline in diet quality and there was also no increase in intake of alcohol or caffeine reported over the first year of medical school in our sample.

Our results show that overall diet quality among 195 participating medical students was above the national average of 58 [20], but still below desirable levels. Participant reported average intake of fiber of is below the recommended 25–38 g/day [21]. Fiber is a shortfall nutrient in the US [19], and medical students are also not getting the recommended minimal daily amount. Average sodium intake was well above recommendations [19], while potassium intake was below recommended levels [19]. These findings are important for two main reasons: first, diet quality is less than desirable among medical students, coupled with the high stress and limited sleep typical of medical school puts students at risk for preventable chronic disease. Healthy habits tend to decline in the first year of medical school due to the pressures and time limitations of engaging in a rigorous curriculum [22]. Our finding that diet did not significantly decline during the first year of medical school suggests that a culinary medicine and nutrition curriculum could help prevent these declines. Secondly, evidence from studies among medical students [18, 23] and physicians [24, 25] suggest that personal lifestyle habits of health professionals influence provider counseling for patients on a healthy lifestyle [26]. Therefore, educating future physicians not just on nutritional biochemistry and evidence-based lifestyle interventions, but also providing education on food purchasing, preparation and practical information for improving personal diets are integral to training physicians likely to counsel on these topics.

Attendance at all culinary medicine sessions was approximately 92% for the first cohort and 88% for the second cohort. Attendance was excellent for most of the year and declined in April and May as students began studying for their COMLEX and STEP exams. Students were aware most of the topics would not be tested on board exams and the content from CMW held a low weight on their course examinations. There was no penalty for missing CMW sessions, yet students rarely missed sessions until the late spring. Students were able to eat together at the end of each session and shared that the class reduced their stress levels. They received feedback and reinforcement of their knowledge through the TBL readiness assurance process and applied what they learned in the teaching kitchen.

Nutrition and culinary medicine sessions were facilitated by a nutrition faculty member and a Registered Dietitian Nutritionist. This gave students the opportunity to learn from healthcare providers who would be part of a future interdisciplinary healthcare team. Dietetic interns training at a local university attended sessions and functioned as Teaching Assistants. They also shared cases from their own clinical training and answered questions for the medical students on nutrition topics important to them personally and of professional interest. Other faculty members often stopped by the sessions to sample recipes, talk to students, or just observe the culinary medicine sessions. One faculty member reported that she learned more about a student in observing one session than she did in the whole course she taught that the student attended. This attests to the way students are able to be themselves and interact with faculty in a lower stress environment in the CMW. These types of close interactions between faculty and students may help to promote professional identity formation.

Just 26% of respondents took a nutrition class prior to entering medical school and while more than half of survey respondents (*n* = 38) reporting doing their own reading or research related to nutrition, less than half of those have read peer-reviewed nutrition journals or nutrition articles geared toward physicians; instead most respondents reported reading nutrition information in the popular media and in diet books. This suggests that the nutrition information being consumed by future physicians could be fraught with misinformation, without guidance from a nutrition course and nutrition professionals.

Nutrition and culinary medicine curricula are well-suited to team-based learning models and some programs have been redesigned to utilize TBL approaches in the undergraduate setting [27, 28] and in selected medical schools [29, 30]. Hands-on cooking classes are increasingly being taught in medical schools across the country [13, 31, 32], but it does not appear that these programs have implemented team-based learning approaches. A college of pharmacy created a lifestyle modification elective course using TBL and taught it with two different cohorts of second year pharmacy students [33] . Investigators evaluated the impact using pre and post-course surveys and a voluntary course evaluation. Examinations showed improved knowledge of nutrition and lifestyle topics and surveys showed high levels of satisfaction (85%); this was done in a curriculum that was primarily lecture-based.

Colleges of medicine provide inadequate nutrition education to allow future providers to be proficient in having discussions of nutrition and lifestyle with patients. A lack of evidence-based guidance to prepare future doctors has limited progress thus far, but we have agreed upon goals. Future physicians should be prepared to assess nutrition-related problems at the individual and community level, provide basic dietary recommendations to patients, identify patients with or at risk for malnutrition and recognize when to refer to a specialist [10]. Medical schools with lasting/sustainable nutrition programs “thread” it throughout the curriculum from pre-clinical to clinical years aiming for a total of at least 30 h [10] . The medical school curriculum is overloaded, but this program did not take away meaningfully from other coursework and integrated skills and topics that students would see in classes and on national board exams. A review of USMLE step exam questions from 1989 to 1993 found a reasonable amount (11%) of questions dedicated to testing nutrition knowledge, but a dearth of questions related to prevention, nutrition support and malnutrition, with an overemphasis on vitamin deficiencies [34]. A review published in 2015 found that the STEP exam preparatory materials contained many references to vitamin and minerals deficiencies, with few references to prevention or diet-related disease management [35]. An analysis of board examinations in Germany found that < 1% of questions were devoted solely to testing nutrition knowledge and 2% included some nutrition-related topic [36]. This is clearly an area of needed improvement to move nutrition in medical education forward.

A recent report from three institutions utilizing culinary medicine training (at the undergraduate and graduate medical education levels) [37] called for additional research into class format and outcome measures in order to create best practices for implementation of culinary medicine. We contribute this work to the evidence-base, however, this study had several limitations. We did not utilize incentives for completion of surveys as they were administered during class time and there was a low response rate on optional and follow up surveys. Poor responses are common among surveys of medical students, and this study was complicated by rapidly changing restrictions due to a global pandemic. Comparisons between baseline and end of year 1 data should be interpreted with caution due to the smaller sample size that completed both diet assessments and surveys. Diet assessment tools are prone to bias due to poor recall or social desirability and limited ability of respondents to estimate portion sizes. The FFQ used in this study uses images to assist with portion size estimation, which helps to mitigate some error [38]. Students received a study ID and were made aware that their data would be anonymized. The strengths of this study were use of a tested curriculum (HmF), use of a validated tool for data collection (FFQ), and data collection at two time points.

In conclusion, nutrition and diet are important components of preventive care and should be integrated into medical education. Including dietetic interns in training is free of cost and of benefit to all learners. Teaching nutrition in a student friendly, interactive way, was effective, beneficial, and this strategy could be used for teaching other topics, particularly those important for the training of future healthcare providers that may not be tested extensively on board examinations.

## Data Availability

The datasets used and analyzed during the current study are available from the corresponding author on reasonable request.
